# LncRNA LINC00342 promotes gastric cancer progression by targeting the miR-545-5p/CNPY2 axis

**DOI:** 10.1186/s12885-021-08829-x

**Published:** 2021-10-30

**Authors:** Run Liu, Xianwu Yang

**Affiliations:** Department of Gastroenterology, The Shijiazhuang People’s Hospital, 365 Jianhuanan street, Yuhua District, Shijiazhuang, 050000 Hebei China

**Keywords:** Gastric cancer, LINC00342, Tumorigenesis

## Abstract

**Background:**

This study aimed to explore the role and underlying molecular mechanisms of long non-coding RNA (lncRNA) LINC00342 in gastric cancer (GC).

**Methods:**

The expression of LINC00342 in GC tissues was evaluated by Quantitative reverse transcription polymerase chain reaction (qRT-PCR). Silencing of LINC00342 was conducted to investigate the effect of LINC00342 in vitro and in vivo. The underlying molecular mechanisms of LINC00342 were determined by dual luciferase reporter assay, Western blotting analysis and rescue experiments. Biological functions of LINC00342 were evaluated by cell counting kit-8 (CCK-8) assay, colony formation assay, wound healing assay and Transwell assays. In addition, a tumor model was used to verify the effect of LINC00342 in tumorigenesis in vivo.

**Results:**

LINC00342 was significantly upregulated in GC tissues and cell lines. Silencing of LINC00342 efficiently inhibited proliferation, migration and invasion of AGS cells in vitro, and also suppressed the tumorigenesis of GC in vivo. Functional experiments showed that LINC00342 regulated the expression of canopy fibroblast growth factor signaling regulator 2 (CNPY2) by competitively sponging miR-545-5p. Rescue experiments showed that inhibition of miR-545-5p and overexpression of CNPY2 significantly reversed cell phenotypes caused by silencing of LINC00342.

**Conclusion:**

LINC00342 plays a potential oncogenic role in GC by targeting the miR545-5p/CNPY2 axis, and might act as a novel therapeutic target for GC.

## Background

Gastric cancer (GC) is one of the most aggressive malignancies and leading to a significantly increasing mortality in the world [1]. In the past decades, GC has becoming a major global health problem and represents the third most frequent reason for cancer-related deaths [2]. Although progressions have been made in the diagnosis and therapy for GC with surgical techniques and/or adjuvant chemotherapy, the prognosis of GC patients is still poor, and more than 80% of patients are diagnosed at advanced stages [3]. Therefore, it is urgent to develop more effective biomarkers for the diagnosis and treatment of GC.

Long non-coding RNAs (lncRNAs), a class of ncRNAs with more than 200 nucleotides in length [4], have shown extensive biological functions in the pathogenesis of human cancers [5, 6]. It has been reported that lncRNAs function as competitive endogenous RNAs (ceRNAs) and then compete for microRNA (miRNAs) to upregulate the expression of their target genes at the post-transcriptional level [7, 8]. LINC00342 is a newly identified lncRNA and significantly upregulated in non-small cell lung cancer [9]. Studies have investigated the role of LINC00342 in human malignancies. It was reported that LINC00342 promoted cell proliferation and metastasis of non-small cell lung cells by inhibiting its expression [10]. Liu et al.*,* reported that LINC00342 played an oncogenic role in infantile hemangioma and overexpression of LINC00342 accelerated proliferation and reduced apoptosis of hemangioma-derived endothelial cells [11]. Another study found that high expression levels of LINC00342 was related the poor prognosis of colon adenocarcinoma patients and knockdown of LINC00342 suppressed the progression of colon adenocarcinoma [12]. These reports demonstrated the oncogenic role of LINC00342 in cancer, however, the potential functions of LINC00342 in GC remains unclear.

In this study, we found that LINC00342 was significantly upregulated in GC tissues and GC cell lines, and high expression levels of LINC00342 indicated a poor overall survival of GC patients. Specifically, our results revealed that LINC00342 functioned as a ceRNA to regulate the expression of CNPY2 by directly sponging miR545-5p, and promoted cell proliferation, colony formation, migration and invasion in vitro. Moreover, knockdown of LINC00342 efficiently attenuated the tumorigenesis of GC cells using a mouse model in vivo. Taken together, our study provided a novel regulatory axis of LINC00342, miR-545-5p and CNPY2 in GC, indicating that this axis might be a potential therapeutic target for GC treatment.

## Materials and methods

### Patients and tissue specimens

A total of 30 pairs of gastric cancer tissues and matched adjacent normal tissues that were collected by surgical excision from January 2015 to December 2019 and reserved in biological samples library of the Shijiazhuang People’s Hospital were used in this study. Each patient signed the informed consent and all procedures were approved by the Human Ethics Committee of the Shijiazhuang People’s Hospital. All patients were followed up for 5 years to evaluate the overall survival.

### Cell culture

Human gastric cancer cell lines (MKN-28, SGC-7901, HGC-27, NCI-N87, AGS, BGC-823 and FU97) and gastric epithelial cell line (GES-1) were stored at the Digestive Disease Laboratory of the Shijiazhuang People’s Hospital. All cells were cultured in Dulbecco’s modified Eagle’s medium (Gibco, Thermo Fisher Scientific, USA) containing 10% fetal bovine serum (FBS) at 37 °C with 5% CO_2_.

### Cell transfection

Two small interfering RNA targeting LINC00342 (si-LINC00342–1 and − 2) and corresponding negative control (si-NC) were purchased from GenePharma Co. Ltd. (Shanghai, PR, China). MiR-545-5p mimics, NC mimics, miR-545-5p inhibitor and NC inhibitor were also purchased from GenePharma Co. Ltd. To overexpress LINC00342 and CNPY2, the full length of LINC00342 and CNPY2 were synthesized by Sangon Biotech (Shanghai, China) and cloned into pcDNA3.1 (GenePharma, Shanghai, China) vector to generate the overexpressing plasmids, which were named LINC00342 and CNPY2, respectively. And the empty vector pcDNA3.1 was considered as the negative control. Cell transfection for AGS cells was performed using Lipofectamine 3000 (Invitrogen, Carlsbad, CA). The sequences for si-LINC00342 were as follows: si-LINC00342–1: 5′-GAAGAUGCUAACUAGAAUAAC-3′, si-LINC00342–2: 5′-GGAUGAAUUGACAGAAGUAGG-3′ and si-NC: 5′-UUCUCCGAACGUGUCACGU-3′. After transfection for 48 h, the transfection efficiency was confirmed by qRT-PCR and cells were collected for the subsequent experiments.

### The construction of stably knockdown AGS cells

To obtain the LINC00342 stably knockdown AGS cells, the short hairpin RNA against LINC00342 (sh-LINC00342) and negative control (sh-NC) were synthesized by Sangon Biotech (Shanghai, China) and transfected into AGS cells using Lipofectamine 3000. Cells were cultured in DMEM for 72 h and 2 μg/ml puromycin was added to select the stably transfected clones. The stably transfected AGS cells were used to establish the tumor model in vivo.

### Cell proliferation

Cell proliferation was evaluated using cell counting kit-8 kit (CCK-8, Dojindo, Japan). Briefly, approximately 5 × 10^3^ transfected or un-transfected cells AGS cells were seeded into 96-well plates and cultured for 24, 48, 72 and 96 h. At the indicted time, 10 μl of CCK-8 solution was added into each well and incubated for another 2 h. Then the absorbance at 450 nm was detected using a microplate reader (Bio-Rad, Hercules, CA, USA).

### Colony formation assay

Cell proliferation was evaluated using colony formation assay as previously described [13]. In brief, 1 × 10^3^ transfected or un-transfected AGS cells were seeded into 6-well plates. After culturing for 2 weeks, cells were fixed with 10% formaldehyde and then stained with 0.1% crystal violet. The colonies (≥ 40 cells) were photographed and counted under a microscope.

### Wound healing assay

Cell migration was evaluated as previously described [14]. Transfected or un-transfected AGS cells were seeded into 6-well plated and cultured for 24 h. When cell density reached approximately 90%, cell monolayers were scratched using a 100 μl micropipette tip. After culturing for another 48 h, cell migration (at 0 and 48 h) was observed and photographed by using a light microscope (Olympus, Tokyo, Japan).

### Invasion assay

Cell invasive potential was evaluated as previously reported using a modified 24 wells Boyden chamber (Corning, New York, USA), and the 8.0 μm pore polycarbonate filter inserts were coated with Matrigel (BD) [15]. Briefly, FBS-free DMEM containing 1 × 10^3^ transfected or un-transfected AGS cells were added into the upper chamber. The lower chamber was filled with 20% FBS contained DMEM. After 24 h of incubation, the invaded AGS cells in the lower chamber were fixed in 10% formaldehyde and stained with 0.1% crystal violet. The cells were counted and photographed using a light microscope (Olympus, Tokyo, Japan).

### Quantitative reverse transcription polymerase chain reaction (qRT-PCR)

Total RNAs were extracted from tissues or cultured cells using Trizol reagent (Invitrogen, CA, USA). qRT-PCR primers were as follows: LINC00342 F: 5′-CGTTCCAATGTGTTGGGT-3′, R: 5′-TGGGAGGAGGTTGAGATG-3′; miR-545-5p F: 5′-AGCGCGTCAGTAAATGTTTATT-3′, R: 5′-GTTGTTGGTTGGTTGGTTGT-3′; U6 F: 5′-TGCGGGTGCTCGCTTCGGCAGC-3′, R: 5′-GTGCAGGGTCCGAGGT-3′; CNPY2 F: 5′-GACCATGCCCTGCACATATC-3′, R: 5′-TAAAAGGCATTGCCACCATT-3′; GAPDH F: 5′-GCACCGTCAAGGCTGAGAAC-3′, R: 5′-TGGTGAAGACGCCAGTGGA-3′. The relative fold changes of genes were calculated using the 2^−ΔΔCt^ method. U6 and GAPDH were used as the internal control for miR-545-5p and LINC00342/CNPY2, respectively.

### Western blot

Total proteins of tissues or cultured cells were extracted using RIPA lysis buffer. Equal amount of protein samples were separated by 10% SDS-PAGE and transferred into PVDF membranes. Then primary antibody against CNPY2 (Abcam, ab233136, 1:500) and internal reference GAPDH (Abcam, ab9485, 1:1000) were added for incubation with membranes and incubated at 4 °C overnight. Then the membranes were exposed to horseradish peroxidase (HRP)-conjugated IgG for 2 h. The protein bands were visualized using the enhanced chemiluminescence detection kit (ECL, thermo scientific, USA).

### Luciferase reporter assay

The putative binding sites between LINC00342 and miR-545-5p, as well as miR-545-5p and CNPY2 were predicted by StarBase (http://starbase.sysu.edu.cn/index.php). The wild type (WT) and mutant type (MT) 3′-UTR of LINC00342 and CNPY2 containing the putative binding site with miR-545-5p were synthesized by Sangon Biotech (Shanghai, China) and cloned into pmirGLO luciferase vector to generate LINC00342 3′-UTR WT/MT and CNPY2 3′-UTR WT/MT. Then the luciferase reporter vector was co-transfected with miR-545-5p mimics or NC mimics into AGS cells by using Lipofectamine 3000. After 48 h for transfection, the relative luciferase reporter activity was measured using a dual luciferase reporter system.

### Immunofluorescence analysis

Transfected or un-transfected AGS cells were permeabilized in 0.1% Triton X-100 and incubated with anti-CNPY2 antibody (Abcam, ab233136, 1:500) at 4 °C overnight. After washing with PBS, cells were incubated with FITC-conjugated goat anti-rabbit secondary antibody (Abcam, ab6717, 1:1000) for 2 h. Then cell nuclei were stained using DAPI solution (Sangon Biotech, Shanghai, China). Images were captured using a fluorescence microscope.

### In situ hybridization (ISH)

Gastric cancer tissues and adjacent normal tissues were fixed in 4% formaldehyde, dehydrated in gradient ethanol, embedded in paraffin, and cut into 5 μm sections. The Digoxin labeled LINC00342 probe used for ISH was synthesized by Sangon Biotech (Shanghai, China). The slides were processed using the Enhanced Sensitive ISH Detection Kit (POD) (Boster Biotechnology, California, USA), and counterstained in haematoxylin for 90 s. After drying, the slides were photographed using a fluorescence microscope.

### In vivo tumorigenesis

BALB/c nude mice (male, 6-week-old) were used to establish the in vivo model as previously described [13]. In brief, 5 × 10^6^ AGS cells stably transfected with sh-LINC00342 or sh-NC were subcutaneously inoculated into BALB/c nude mice (*n* = 3 per group). The tumor volume was measured using the formula: length × width^2/^2 every 1 week for a total of 5 weeks. Tumors from the two groups were collected and photographed using a digital camera. All animal experiments were approved by the Animal Ethics Committee of the Shijiazhuang People’s Hospital in accordance with the Use Committee for Animal Care.

### Immunohistochemistry (IHC) analysis

The tumors of mice from two groups were cut into 3-μm slices. The antibody against Ki-67 (Abcam, ab15580, 1:500) were used for IHC as previously described [16]. Subsequently, a DAB Substrate Kit (Invitrogen, California, USA) was used to stain the tumor tissues to detect the expression of Ki-67.

### Statistical analysis

All data were presented as means ± standard deviation (SD). Statistical analyses were performed using SPSS 21.0. The diagnostic value of LINC00342 was evaluated by analyzing the ROC curve. The overall survival of GC patients was evaluated using Kaplan-Meier curve followed by log-rank test. Spearman Pearson correlation analysis was used to assess the relationship between the expression levels of LINC00342 and miR-545-5p. Difference between two groups was explored by Student’s t-test. Differences among multiple groups were explored by one-way ANOVA. Correlation between LINC00342 and CNPY2 was analyzed by Linear regression analysis. *P* < 0.05 was considered as the significant threshold.

## Results

### LINC00342 was significantly upregulated in GC tissues

**Fig. 1:**
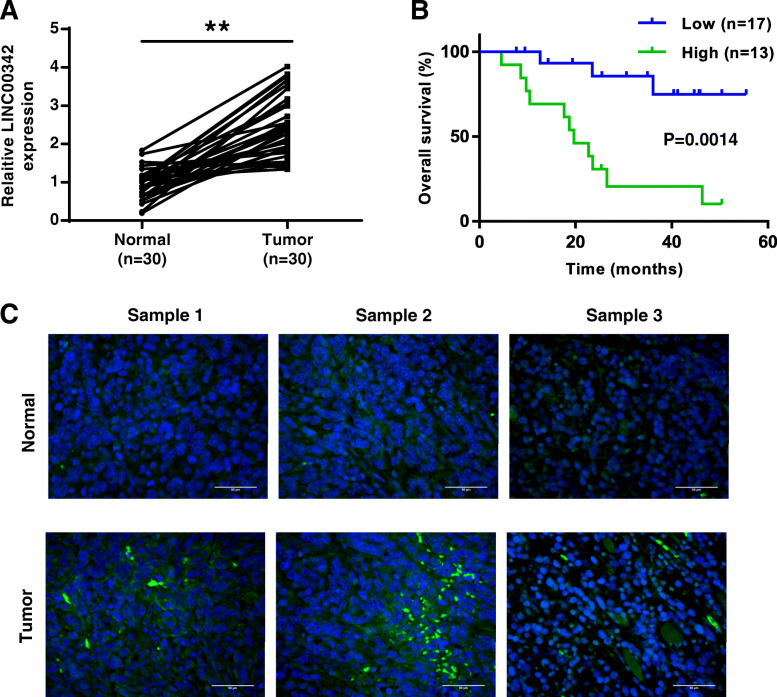
LINC00342 was significantly upregulated in GC tissues. **A** The expression of LINC00342 in GC tissues (*n* = 30) and matched adjacent tissues (*n* = 30) was measured by qRT-PCR. **B** The ROC analysis for the detection of GC. **C** The overall survival of GC patients was evaluated by Kaplan-Meier curve based on the expression of LINC00342 (*n* = 30). **D** The expression of LINC00342 in GC tissues and adjacent tissues was evaluated by using in situ hybridization (ISH) assay (*n* = 3). × 400 magnifications, scale bar = 50 μm. Green color: LINC00342; blue color: nuclei. ** *p* < 0.01. **E** The correlation between LINC00342 and CNPY2 in GC patients’ tissue samples

To explore the role of LINC00342 in GC, the expression of LINC00342 in GC tissues was evaluated by qRT-PCR and the results showed that LIC00342 was significantly upregulated in GC tissues (*n* = 30) compared with that in matched adjacent tissues (*n* = 30) (*p* < 0.01, Fig. 1a). As shown in Fig. 1b, LINC00342 showed high diagnostic value (AUC value = 0.8762; sensitivity = 92.31% and specificity = 86.72%). In addition, all GC patients were divided into two groups based on the expression of LINC00342. Kaplan-Meier curve showed that high expression levels of LINC00342 was associated with poor survival (*n* = 30, *p* < 0.01, Fig. 1c). Meanwhile, the expression of LINC00342 in GC tissues and matched adjacent tissues was evaluated by ISH assay and the results showed that LINC00342 was obviously upregulated in GC tissues compared with that in matched adjacent tissues (Fig. 1d). These results indicate that LINC00342 may act as an oncogene in GC. Furthermore, correlation analysis showed that the expression of LINC00342 was positively correlated with the expression of CNPY2 in GC tissues (Fig. 1e).

Silencing of LINC00342 inhibited the proliferation, migration and invasion of GC cell line AGS.

**Fig. 2:**
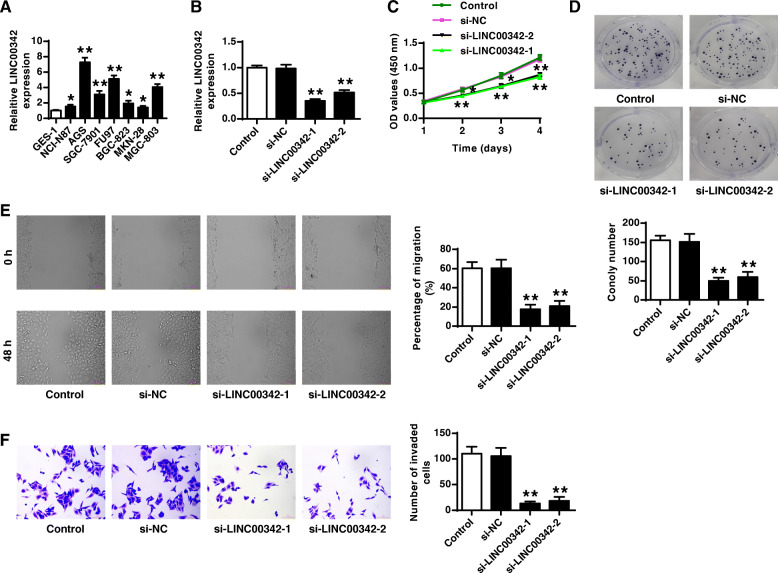
Silencing of LINC00342 inhibited the proliferation, migration and invasion of AGS cells. **A** The expression of LINC00342 in seven GC cell lines was evaluated by qRT-PCR. **B**-**F** Two siRNAs targeting LINC00342 were transfected into AGS cells. **B** The transfection efficiency was detected by qRT-PCR. Cell proliferation was evaluated by CCK-8 assay (**C**) and colony formation assay (**D**). **E** Cell migration was evaluated by wound healing assay. **F** Cell invasion was evaluated by Transwell assay. * *p* < 0.05, ** *p* < 0.01 vs si-NC

To further confirm the high expression levels of LINC00342 in GC, the expression of LINC00342 in 7 GC cell lines was evaluated by qRT-PCR and the results showed that LINC00342 was significantly upregulated in all 7 GC cell lines (all *p* < 0.05) and had the highest expression level in AGS cells (Fig. 2a), which was therefore selected for the subsequent experiments. Two si-RNAs targeting LINC00342 were transfected into AGS cells and it showed that both si-LINC00342–1 and si-LINC00342–2 reduced the expression levels of LINC00342 compared with si-NC (both *p* < 0.01, Fig. 2b). Both Si-LINC00342–1 and si-LINC00342–2 reduced cell viability (both *p* < 0.01) and colony formation (both *p* < 0.01) of AGS cells compared with si-NC (Fig. 2c and d). In addition, both si-LINC00342–1 and si-LINC00342–2 inhibited cell migration (both *p* < 0.01) and invasion (both *p* < 0.01) of AGS cells compared with si-NC (Fig. 2e and f). These results indicated that silencing of LINC00342 inhibited the proliferation, migration and invasion of AGS cells.

### LINC00342 served as a sponge of miR-545-5p

**Fig. 3:**
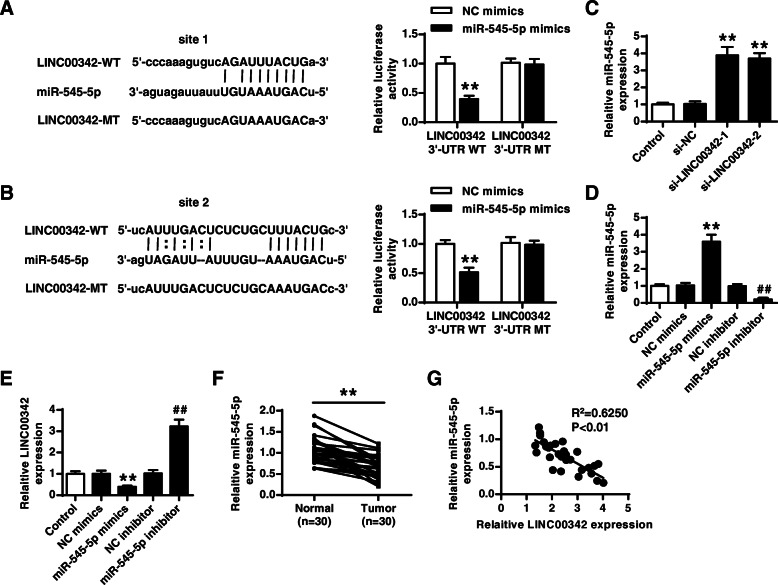
LINC00342 served as a sponge of miR-545-5p. **A** and **B** The putative binding sites between LINC00342 and miR-545-5p was predicted by StarBase, and the relative luciferase activity of LINC00342 WT/MT was detected by dual luciferase reporter system. **C** AGS cells were transfected with two siRNAs targeting LINC00342, and the expression of miR-545-5p was evaluated by qRT-PCR. **D** and **E** AGS cells were transfected with miR-545-5p mimics/NC mimics, or miR-545-5p inhibitor/NC inhibitor. The expression of miR-545-5p (**D**) and LINC00342 (**E**) was evaluated by qRT-PCR. (F) The expression of miR-545-5p in GC tissues (*n* = 30) and matched adjacent tissues (*n* = 30) was measured by qRT-PCR. **G** The correlation between the levels of LINC00342 and miR-545-5p was evaluated by Spearman’s correlation analysis. ** *p* < 0.01 vs NC mimics/si-NC; ## *p* < 0.01 vs NC inhibitor

By using the bioinformatics tool StarBase, we found that there were two putative binding sites between LINC00342 and miR-545-5p (Fig. 3a and b), suggesting that LINC00342 might serve as the sponge for miR-545-5p. To further determine their relationship, luciferase reporter assay was performed, and the results showed that overexpression of miR-545-5p significantly reduced the relative luciferase activity of LINC00342 WT containing both two putative binding sites compared with NC mimics (both *p* < 0.01), but it had no effect on LINC00342 MT (Fig. 3a and b). Meanwhile, both si-LINC00342–1 and si-LINC00342–2 significantly increased the expression levels of miR-545-5p in AGS cells compared with si-NC (both *p* < 0.01, Fig. 3c). Next, miR-545-5p mimics and inhibitor were transfected into AGS cells and the transfection efficiency was confirmed by qRT-PCR (both *p* < 0.01, Fig. 3d). MiR-545-5p mimics significantly reduced the expression levels of LINC00342 compared with NC mimics (*p* < 0.01) and miR-545-5p inhibitor increased the expression levels of LINC00342 compared with NC inhibitor (*p* < 0.01, Fig. 3e). The expression levels of miR-545-5p in GC tissues were evaluated by qRT-PCR and the results showed that miR-545-5p was significantly downregulated in GC tissues (*n* = 30) compared with that in matched adjacent tissues (*p* < 0.01, Fig. 3f). In addition, Spearman’s rank correlation analysis showed that the expression of LINC00342 was negatively correlated with the expression of miR-545-5p in GC tissues (*n* = 30, *p* < 0.01, Fig. 3g). These results suggested that LINC00342 served as a sponge of miR-545-5p.

### MiR-545-5p mimics inhibited the proliferation, migration and invasion of GC cell line AGS possibly through CNPY2

**Fig. 4:**
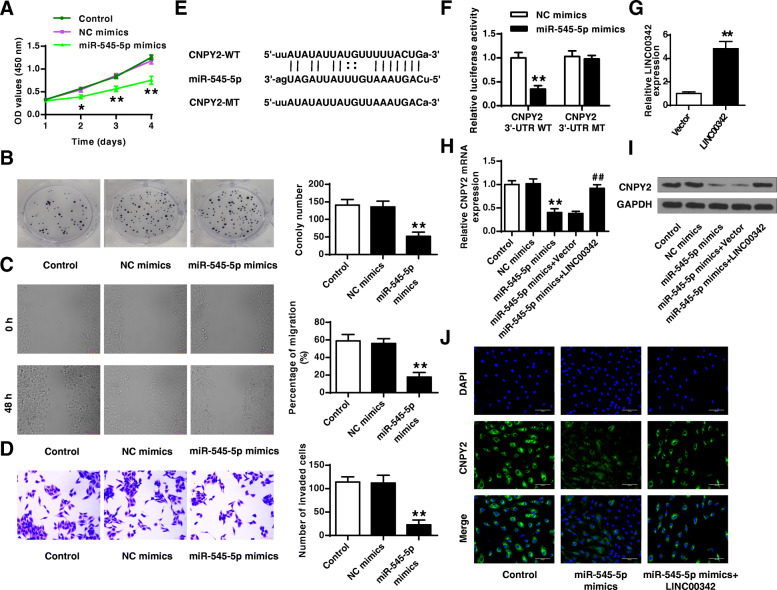
MiR-545-5p mimics inhibited the proliferation, migration and invasion of GC cell line AGS might through CNPY2. **A**-**D** AGS cells were transfected with miR-545-5p mimics or NC mimics. Cell proliferation was evaluated by CCK-8 assay (**A**) and colony formation assay (**B**). **C** Cell migration was evaluated by wound healing assay. **D** Cell invasion was evaluated by Transwell assay. **E** The putative binding site between miR-545-5p and CNPY2 was predicted by Targetscan. **F** The relative luciferase activity of CNPY2 WT/MT was detected by dual luciferase reporter system. **G** AGS cells were transfected with overexpressing plasmid (LINC00342) and the empty vector, and the transfection efficiency was confirmed by qRT-PCR. (H-J) AGS cells were transfected with miR-545-5p mimics, or co-transfected with miR-545-5p mimics and overexpressing plasmid (LINC00342). The expression of CNPY2 was evaluated by qRT-PCR (**H**) and western blot (**I**). **J** The expression of CNPY2 was evaluated by immunofluorescence using anti-CNPY2 antibody. × 200 magnification, scale bar = 100 μm. * *p* < 0.05, ** *p* < 0.01 vs NC mimics/the empty vector; ## *p* < 0.01 vs miR-545-5p mimics + the empty vector

To explore the role of miR-545-5p in GC, AGS cells were transfected with miR-545-5p mimics or NC mimics, and cell proliferation was evaluated. The results showed that overexpression of miR-545-5p significantly inhibited cell viability (*p* < 0.01) and colony formation (*p* < 0.01) of AGS cells compared with NC mimics (Fig. 4a and b). Meanwhile, overexpression of miR-545-5p also inhibited cell migration (*p* < 0.01) and invasion (*p* < 0.01) of AGS cells compared with NC mimics (Fig. 4c and d). The potential targets of miR-545-5p were predicted by Starbase, and the results showed that CNPY2 might be a target of miR-545-5p (Fig. 4e). Overexpression of miR-545-5p significantly reduced the relative luciferase activity of CNPY2 WT compared with NC mimics (*p* < 0.01) but had no effect on CNPY2 MT (Fig. 4f). Overexpressing plasmid for LINC00342 (LINC00342) and the empty vector control were transfected into AGS cells, and the transfection efficiency was confirmed by qRT-PCR (*p* < 0.01, Fig. 4g). Rescue experiments were performed in AGS cells to evaluate the effects of LINC00342 and miR-545-5p on the expression of CNPY2, and the results showed that miR-545-5p mimics reduced the expression levels of CNPY2 compared with NC mimics (*p* < 0.01), overexpression of LINC00342 obviously reversed miR-545-5p mimics-induced inhibition on the expression of CNPY2 (*p* < 0.01, Fig. 4h and i). The results of immunofluorescence showed that miR-545-5p mimics obviously reduced the number of CNPY2-positive cells, and overexpression of LINC00342 obviously relieved miR-545-5p mimics-induced inhibition on the expression of CNPY2 (Fig. 4j). These results indicated that miR-545-5p mimics inhibited the proliferation, migration and invasion of GC cell line AGS possibly through CNPY2.

### MiR-545-5p mediated the effect of LINC00342 on AGS cells

**Fig. 5:**
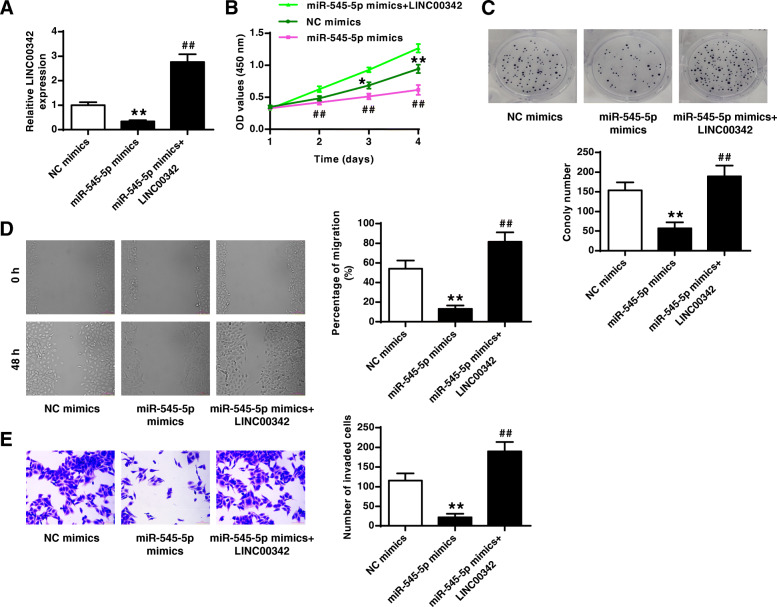
MiR-545-5p mediated the effect of LINC00342 in AGS cells. AGS cells were transfected with NC mimics, miR-545-5p mimics, or co-transfected with miR-545-5p mimics and overexpressing plasmid (LINC00342). **A** The transfection efficiency was confirmed by qRT-PCR. **B** and **C** Cell proliferation was evaluated by CCK-8 assay (**B**) and colony formation assay (**C**). **D** Cell migration was evaluated by wound healing assay. **E** Cell invasion was evaluated by Transwell assay. ** *p* < 0.01 vs NC mimics; ## *p* < 0.01 vs miR-545-5p mimics

To explore whether miR-545-5p mediated the role of LINC00342 in GC, AGS cells were transfected with miR-545-5p mimics and overexpressing plasmid (LINC00342), and the transfection efficiency was confirmed by qRT-PCR (*p* < 0.01, Fig. 5a). Overexpression of miR-545-5p inhibited cell viability, colony formation, migration and invasion of AGS cells compared with NC mimics (all *p* < 0.01), while the inhibitory effects of miR-545-5p mimics were significantly reversed by the co-transfection of miR-545-5p mimics and LINC00342 (all *p* < 0.01, Fig. 5b-e). These results demonstrated that miR-545-5p mediated the effect of LINC00342 on AGS cells.

### Overexpression of CNPY2 reversed the protective effects of si-LINC00342 on AGS cells

**Fig. 6:**
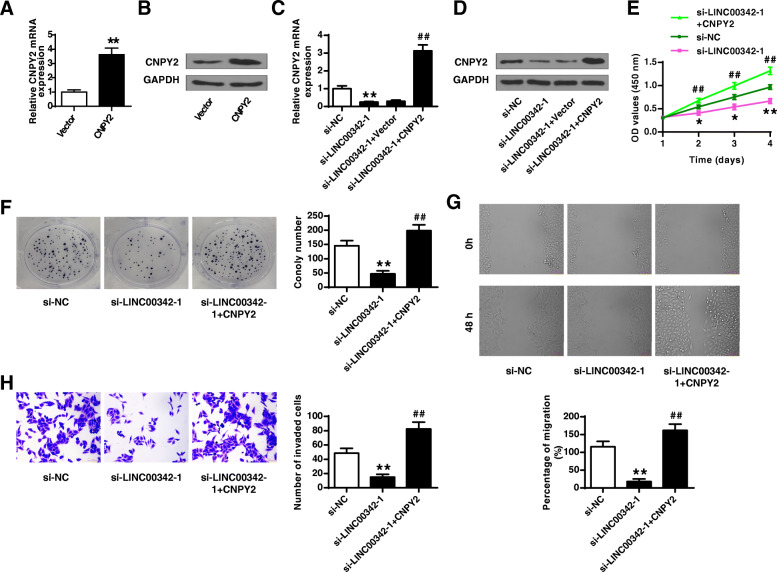
Overexpression of CNPY2 reversed the protective effects of si-LINC00342 in AGS cells. **A** and **B** AGS cells were transfected with the overexpressing plasmid (CNPY2) and the empty vector. The transfection efficiency was confirmed by qRT-PCR (**A**) and western blot (**B**). **C**-**H** AGS cells were transfected with si-LINC00342–1, si-NC, or co-transfected with si-LINC00342–1 and the overexpressing plasmid (CNPY2). The expression of CNPY2 was evaluated by qRT-PCR (**C**) and western blot (**D**). Cell proliferation was evaluated by CCK-8 assay (**E**) and colony formation assay (**F**). **G** Cell migration was evaluated by wound healing assay. **H** Cell invasion was evaluated by Transwell assay. ** *p* < 0.01 vs si-NC; ## *p* < 0.01 vs si-LINC00342–1

Next, rescue experiments were performed to investigate whether CNPY2 mediated the role of LINC00342 in GC. AGS cells were transfected with the overexpressing plasmid (CNPY2) and the empty vector, and the results showed that overexpression of CNPY2 significantly increased the expression levels of CNPY2 compared with the empty vector (*p* < 0.01, Fig. 6a and b). In addition, overexpression of CNPY2 obviously relieved the inhibitory effects of si-LINC00342–1 on the expression of CNPY2 in AGS cells (*p* < 0.01, Fig. 6c and d). Moreover, the inhibitory effects of si-LINC00342–1 on cell viability, colony formation, migration and invasion were obviously eliminated by co-transfection of si-LINC00342–1 and the overexpressing plasmid (CNPY2) (all *p* < 0.01, Fig. 6e-h). These results indicated that overexpression of CNPY2 reverses the protective effects of si-LINC00342 on cell proliferation, migration and invasion in vitro.

### Knockdown of LINC003421 efficiently suppressed the tumorigenesis of GC by regulating the miR-545-5p/CNPY2 axis in vivo

**Fig. 7:**
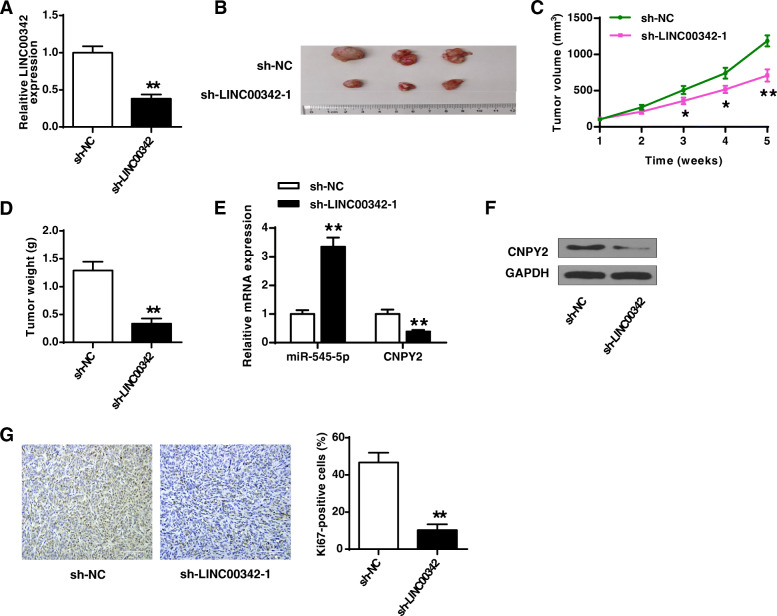
Knockdown of LINC003421 efficiently suppressed the tumorigenesis of GC by regulating miR-545-5p/CNPY2 axis in vivo. The tumor model was established by the subcutaneous inoculation with stably sh-LINC00342–1 or sh-NC transfected AGS cells. **A** The expression of LINC00342 in tumor tissues was evaluated by qRT-PCR. **B** The representative images of tumors in two groups. **C** Tumor volume. **D** Tumor weight. **E** The expression of miR-545-5p and CNPY2 in tumor tissues was evaluated by qRT-PCR. **F** The expression of CNPY2 in tumor tissues was evaluated by western blot. **G** Cell proliferation in tumor tissues was evaluated by IHC assay using anti-Ki67 antibody. × 200 magnification, scale bar = 100 μm. Gray color: Ki67; Blue color: nuclei. * *p* < 0.05, ** *p* < 0.01 vs sh-NC

Finally, the tumor model was constructed by the subcutaneous inoculation with stably sh-LINC00342–1 or sh-NC transfected AGS cells. The knockdown efficiency of sh-LINC00342 was confirmed by qRT-PCR (*p* < 0.01, Fig. 7a). Moreover, knockdown of LINC00342 significantly reduced tumor volume (*p* < 0.05) and tumor weight (*p* < 0.01) compared with sh-NC (Fig. 7b-d). The expression of miR-545-5p and CNPY2 in tumor tissues was assessed and the results showed that knockdown of LINC00342 significantly increased the expression levels of miR-545-5p (*p* < 0.01) and reduced the expression levels of CNPY2 (*p* < 0.01) in tumor tissues compared with negative control sh-NC (Fig. 7e and f). In addition, IHC assay results showed that knockdown of LINC00342 obviously reduced the number of Ki-67 positive cells in tumor tissues compared with sh-NC (*p* < 0.01, Fig. 7g). These results demonstrated that knockdown of LINC003421 efficiently suppressed the tumorigenesis of GC by regulating the miR-545-5p/CNPY2 axis in vivo.

## Discussion

LINC00342 is a potential oncogenic lncRNA that has been characterized in non-small cell lung cancer [10], infantile hemangioma [11], and colon adenocarcinoma [12]. In the present study, we firstly reported that LINC00342 potentially played an oncogenic role in GC. We also showed that the function of LINC00342 in GC was partially mediated by the miR-545-5p/CNPY2 axis. Our study extended the understanding of lncRNAs in GC.

Except for LINC00342, there were a large number of abnormally expressed lncRNAs involved in the pathogenesis and prognosis of GC [17]. For example, overexpression of HOXA11-AS aggravates cell cycle progression and metastasis of GC cells in vitro [18]. LINC02532 promotes cell proliferation, migration and invasion of GC cells, and may be a promising target for therapy and prognosis [19]. Extensive studies have identified various lncRNAs that potentially participate in the progression of GC [17], and the functions of many lncRNAs that have been well studied in other cancers are not clear in GC. LINC00342 has been demonstrated to affect the progression of several types of human cancer, but its function and underlying molecular mechanisms remains unclear. In this study, we revealed that GC patients showed obviously higher expression levels of LINC00342, which also represented a poor prognosis of GC patients. Despite of the oncogenic role of LINC00342 in other cancers, we confirmed its oncogenic role in GC, suggesting that LINC00342 might be a new target for drug development in GC treatment.

Increasing evidence has revealed that lncRNAs often exert their crucial regulatory functions by adsorbing miRNAs to reduce their expression levels [20, 21]. In GC, PAPAS aggravates GC progression by directly regulating miR-188-5p [22]. CASC11 promotes cell proliferation, migration and invasion of GC cells in vitro by regulating cell cycle pathway via targeting miR-340-5p [23]. Here, we identified that LINC00342 acted as a ceRNA of miR-545-5p by binding to two sites at 3′-UTR of miR-545-5p. One previous study also reported that miR-545-5p played an essential role in the regulatory mechanism of LINC00342 in colon adenocarcinoma [12]. The previous study demonstrated that LINC00342 acted as a ceRNA of miR-545-5p by only one site, and our study identified that there were two putative binding sites between them. Luciferase reporter assay further demonstrated that the two sites were both important for the interaction between LINC00342 and miR-545-5p. Furthermore, rescue experiments revealed that overexpression of miR-545-5p significantly attenuated the inhibitory effects of si-LINC00342 on cell phenotypes in vitro.

CNPY2 is key regulator of different types of human cancer such as renal cell carcinoma [24], colorectal cancer [25], non-small cell lung cancer [26], and lung adenocarcinoma [27]. Here, CNPY2 was found to be a direct target of miR-545-5p, and LINC00342 negatively regulated the expression of CNPY2 by sponging miR-545-5p. Moreover, overexpression of CNPY2 obviously reversed the inhibitory effects of si-LINC00342 on AGS cell proliferation, migration and invasion in vitro. A previously study demonstrated that CNPY2 participated in the regulation of miR-30a-3p in the proliferation, migration and invasion of lung adenocarcinoma cells [27]. In the last decades, CNPY2 was found to regulate the progression of human diseases mainly though modifying various downstream signaling pathways including p53 in colorectal cancer [25], NF-κB pathway in non-small cell lung cancer [28], as well as the PERK signaling pathway in myocardial ischemia reperfusion injury [29]. Of which, the p53 signaling pathway and the NF-κB signaling pathway were closely associated with the proliferation and metastasis of GC [30, 31]. Whether one or more relevant signing pathway(s) mediated the role of the LINC00342/miR-545-5p/CNPY2 axis in GC progression needs to be investigated in future studies.

The xenograft tumor model was a widely used approach to explore the role of lncRNAs/miRNAs/mRNAs in the tumorigenesis in vivo [32, 33]. To confirm the role of LINC00342 in GC, an animal model was established, and the in vivo experiments further confirmed the oncogenic function of knockdown of LINC00342 in GC, and the miR-545-5p/CNPY2 axis participated in the regulation of LINC00342 in GC.

Previous studies have found that ncRNAs have critical roles in human disorders by regulating apoptosis and oxidative stresses through multiple pathways, such as the ERK/MAPK pathway and nuclear factor-κB (NF-κB) pathway [34–36]. In our study, we found that LINC00342 and miR-545-5p regulated cell proliferation, migration, and invasion in gastric cancer by regulating CNPY2. A previous study found that CNPY2 repressed cisplatin-induced apoptosis in lung cancer by activating the NF-κB pathway [28]. Therefore, we suspected that LINC00342 and miR-545-5p may also affect cancer cell apoptosis in gastric cancer by regulating the CNPY2/NF-κB pathway, which will be investigated in our future study.

In summary, our study demonstrated that the LINC00342/miR-545-5p/CNPY2 axis participated in the progression of GC, providing new insight into the post-transcriptional regulation mechanism of lncRNAs in GC.

## Data Availability

The data was available form corresponding author upon reasonable request.
